# Melatonin attenuates doxorubicin‐induced cardiotoxicity through preservation of YAP expression

**DOI:** 10.1111/jcmm.15057

**Published:** 2020-02-18

**Authors:** Hai‐ru Li, Chao Wang, Ping Sun, Dan‐dan Liu, Guo‐qing Du, Jia‐wei Tian

**Affiliations:** ^1^ Department of Ultrasound The Second Affiliated Hospital of Harbin Medical University Harbin China; ^2^ Key Laboratories of Myocardial Ischemia Mechanism and Treatment Harbin Medical University, Ministry of Education Harbin China; ^3^ Department of Cardiology The Second Affiliated Hospital of Harbin Medical University Harbin China

**Keywords:** apoptosis, cardiotoxicity, doxorubicin, melatonin, YAP

## Abstract

There are increasing concerns related to the cardiotoxicity of doxorubicin in the clinical setting. Recently, melatonin has been shown to exert a cardioprotective effect in various cardiovascular diseases, including cardiotoxic conditions. In this study, we examined the possible protective effects of melatonin on doxorubicin‐induced cardiotoxicity and explored the underlying mechanisms related to this process. We found that in vitro doxorubicin treatment significantly decreased H9c2 cell viability and induced apoptosis as manifested by increased TUNEL‐positive cells, down‐regulation of anti‐apoptotic protein Bcl‐2, as well as up‐regulation of pro‐apoptotic protein Bax. This was associated with increased reactive oxygen species (ROS) levels and decreased mitochondrial membrane potentials (MMP). In vivo, five weeks of doxorubicin treatment significantly decreased cardiac function, as evaluated by echocardiography. TUNEL staining results confirmed the increased apoptosis caused by doxorubicin. On the other hand, combinational treatment of doxorubicin with melatonin decreased cardiomyocyte ROS and apoptosis levels, along with increasing MMP. Such doxorubicin‐melatonin co‐treatment alleviated in vivo doxorubicin‐induced cardiac injury. Western Blots, along with in vitro immunofluorescence and in vivo immunohistochemical staining confirmed that doxorubicin treatment significantly down‐regulated Yes‐associated protein (YAP) expression, while YAP levels were maintained under co‐treatment of doxorubicin and melatonin. YAP inhibition by siRNA abolished the protective effects of melatonin on doxorubicin‐treated cardiomyocytes, with reversed ROS level and apoptosis. Our findings suggested that melatonin treatment attenuated doxorubicin‐induced cardiotoxicity through preserving YAP levels, which in turn decreases oxidative stress and apoptosis.

## 1 INTRODUCTION

Doxorubicin (Dox) is an effective anti‐neoplastic medication, widely used in the treatment of solid cancers and haematological malignancies.^1^ However, it has limited clinical use owing to its acute and chronic cardiotoxicity, which mainly manifests in the form of left ventricular dysfunction and ultimate heart failure. In fact, a study conducted by Tan et al showed that in a cohort of women treated with anthracyclines and trastuzumab, left ventricular end‐diastolic and end‐systolic volumes increased, while ejection fraction, strain and strain rate decreased at the end of treatment compared with baseline. There, no recovery was present during >2 years follow‐up.^2^, ^3^ It has been demonstrated that Dox induces cardiomyocyte toxicity and cell death through a variety of mechanisms, the most prominent being the production of excess reactive oxygen species (ROS).^4^ Cardiac tissue contains abundant mitochondria, which are essential for cardiomyocytes to sustain sufficient ATP production for contractile function and cell survival.^5^, ^6^ Dox specifically targets mitochondria, where its accumulation there results in the destruction of mitochondrial membrane structure, interference with oxidative respiration, and reduction of mitochondrial membrane potential (MMP), all of which eventually leads to cardiomyocyte apoptosis.^7^ The apoptotic effects of Dox were further proved by studies showing that Dox treatment is capable of significantly increasing the expression of pro‐apoptotic protein Bax, as well as decreasing the expression of anti‐apoptosis protein Bcl‐2.^8^, ^9^, ^10^

Melatonin (Mel), endogenously‐produced by the pineal gland of mammals, has recently been implicated in the modulation of various cardiovascular diseases.^11^ Studies have shown that Mel alleviates post‐infarct cardiac remodelling and dysfunction through up‐regulating autophagy, decreasing apoptosis and modulating mitochondrial integrity and biogenesis.^11^ Furthermore, there is evidence suggesting that Mel is able to reduce the infarct area, sustain myocardial function and suppress cardiomyocyte death during cardiac ischaemia‐reperfusion injury.^12^, ^13^ Mel also abrogates diabetic cardiomyopathy, by reducing ROS level and rescuing impaired mitophagy activity.^14^, ^15^ Additional studies also showed Mel being involved in alleviating mitochondrial oxidative damage and apoptosis caused by Dox in cardiomyocytes.^2^, ^16^ However, the exact mechanism mediating this protective effect of Mel on Dox‐induced cardiotoxicity remains unclear.

YAP (Yes‐associated protein, also known as YAP1) is the downstream effector of the Hippo signalling pathway, where it participates in diverse physiological and pathological processes related to heart development, apoptosis, hypertrophy, autophagy, angiogenesis and basal homoeostasis.^17^ Inactivation of YAP increases cardiomyocyte apoptosis and fibrosis, as well as aggravating cardiac dysfunction after a myocardial infarction (MI).^18^ Conversely, cardiac‐specific YAP activation after MI has been demonstrated to mitigate myocardial injury and improve cardiac function, the latter being associated with enhanced cardiomyocyte survival via encouraging a less mature cardiac gene expression profile. This profile entails the stimulation of cell cycle genes.^19^ Human ischaemic and non‐ischaemic heart failure activates the Hippo pathway, while its inactivation reverses systolic heart failure after MI.^20^ Another YAP‐related activity is the modulation of antioxidant capacity, where YAP inactivation suppresses FoxO1 activity and decreases antioxidant gene expression, thus aggravating ischaemia‐reperfusion induced heart injury.^21^

The present study is aimed to investigate whether Mel can protect cardiomyocytes from Dox‐induced oxidative stress injury and apoptosis, as well as underlying mechanism and mediators involved if such protection actually took place. We showed that Mel treatment attenuated Dox‐induced cardiotoxicity through preserving YAP levels, which decreased oxidative stress and apoptosis.

## 2 MATERIALS AND METHODS

### 2.1 Drugs and reagents

Doxorubicin was purchased from Meilunbio (A0520AS). Melatonin was purchased from Yuanye biotechnology (B21269). Dimethyl sulfoxide (DMSO) and Trizol Reagent were purchased from Invitrogen. Primary antibodies against YAP were purchased from Cell Signaling Technology (14074S). Antibodies against Bax and Bcl‐2 were purchased from Proteintech (50599‐2‐lg and 12789‐1‐AP). Antibodies against β‐actin, as well as goat anti‐rabbit and goat anti‐mouse secondary antibodies were purchased from Zhongshan Company. Cell Counting Kit‐8 (CCK‐8) was provided by Dojindo. Terminal deoxynucleotidyl transferase dUTP nick‐end labelling (TUNEL) was purchased from Roche Diagnostics. The 2′,7′‐dichlorofluorescein diacetate (DCFH‐DA), and mitochondrial membrane potential assay kit with JC‐1, were purchased from Beyotime Institute of Biotechnology. Histostain™‐SP Kits (SPN‐9002) and DAB (ZLI‐9017) were purchased from Zhongshan Company. FITC‐labelled wheat germ agglutinin(WGA) was purchased from Sigma (L4895).

### 2.2 Cytotoxicity of Mel and Dox

H9c2 cells were obtained from the BeNa Culture Collection (Beijing, China) and maintained in DMEM supplemented with 10% FBS in a humidified atmosphere of 5% CO_2_ and 95% O_2_ at 37°C. To test the toxicity of Dox and Mel, the cells were plated in 96‐well plates for 24 hours, at a density of 5 × 10^3^ per well, before treatment with different concentrations of Mel (0‐200 μmol/L) or Dox (0.25‐5 μmol/L). Mel and Dox stock solutions were prepared in 0.1% DMSO and diluted in culture media immediately prior to experiments. Control group cells were treated with 0.1% DMSO. Two hours before the end of incubation, CCK‐8 (10 μL) was added to 100 μL medium in each well, and then incubated at 37°C for 2 hours, before measuring absorbance at 450 nm with a microplate Reader (SpectraMax 190, Molecular Device). Cell viability was expressed as the ratio of OD values of experimental wells to that of control wells.

To investigate whether Mel has a protective effect on Dox‐induced cardiotoxicity, H9c2 cells were pre‐treated with different concentrations of Mel (0‐100 μmol/L) for 24 hours after cell inoculation, then incubated with Dox for 24 hours.

### 2.3 siRNA transfection and in vitro experimental groups

Commercially available rat YAP and control siRNA were purchased from RiboBio Co. LTD. H9c2 cells were plated on 6‐well plates, at 2 × 10^5^ cells per well, in 2 mL of antibiotic‐free normal growth medium supplemented with 10% FBS. After the cells reached 30%‐50% confluence, they were transfected with either control or targeted siRNA duplex, according to the kit instructions. Cells were harvested for further experiments after 48 hours transfection.

Cells were assigned into four groups in the initial experiment: (i) Control; (ii) Dox (1 μmol/L) treatment; (iii) Mel (10 μmol/L) treatment; (iv) Mel and Dox co‐treatment (Mel+Dox). In the subsequent experiments, cells were then assigned to the following six groups: (i) Control; (ii) Dox; (iii) Mel; (iv) Mel+Dox; (v) Mel+Dox+siNC (control siRNA); (vi) Mel+Dox+siYAP (target siRNA).

### 2.4 Analysis of cell apoptosis by TUNEL assay

Apoptosis of H9c2 cells and mice heart were detected by the TUNEL assay, according to the manufacturer's instructions. H9c2 cells were inoculated in 48 well plates, at 2.5 × 10^4^ per well. In vivo paraffin sections were cut to 5 μm thickness, and images obtained under fluorescence microscopy (Olympus). Apoptotic index was expressed as the ratio of TUNEL‐positive (green) to DAPI‐positive myocytes (blue).

### 2.5 Analysis of ROS

DCFH‐DA staining method was used to measure the generation of ROS. Pre‐treated H9c2 cells (5 × 10^4^) were inoculated in 48‐well plates and incubated in darkness with DCFH‐DA (10 μmol/L) for 25 minutes at 37°C. Fluorescent intensity was immediately measured under fluorescence microscopy (Olympus), with excitation at 488 nm and emission at 522 nm.

### 2.6 Determination of mitochondrial membrane potential

A mitochondrial membrane potential assay kit utilizing JC‐1 (Beyotime) was applied to measure mitochondrial membrane potential (MMP). The experimental procedure was operated according to the manufacturer's instructions. At high MMP, JC‐1 accumulated in the mitochondrial matrix, forming polymers (J‐aggregates), which yielded a red fluorescent light. At low MMP, JC‐1 dissociated into monomers, which were unable to attach to the mitochondrial matrix. These monomers emitted a green fluorescent light. Fluorescent intensity was immediately measured under fluorescence microscopy (Olympus). MMP was quantified as the ratio of mean green fluorescent intensity (monomer)/mean red fluorescent intensity (polymer).

### 2.7 Real‐time quantitative polymerase chain reaction (RT‐qPCR)

Total RNA was extracted with TRIzol reagent, which was then reverse transcribed into complementary DNA (cDNA) with Transcriptor First Strand cDNA Synthesis Kit (Roche, Switzerland). Real‐time qPCR was carried out using SYBR® Green real‐time PCR Master Mix (Roche), normalized to house‐keeping gene GAPDH. For rat YAP, the forward primer was 5′‐ACCATAAGAACAAGACCACATCC‐3′, and reverse primer 5′‐TTCAATCGCAGCCTCTCCTT‐3′. Rat GAPDH forward primer was 5′‐ATGCCATCACTGCCACTCA‐3′, and reverse primer 5′‐CCTGCTTCACCACCTTCTTG‐3′. Rat connective tissue growth factor (CTGF) had the forward primer 5′‐CCGATGGCGAGATCATGAAA‐3′ and reverse primer 5′‐CGCCATGTCTCCATACATCTT‐3′. Rat parkin RBR E3 ubiquitin protein ligase (Park2) had the forward primer 5′‐GACCAGCTGCGAGTGATTT‐3′ and reverse primer 5′‐TCCTCTGTGGTCTCTGTACTATG‐3′. For rat baculoviral IAP repeat containing 5 (birc5), the forward primer was 5′‐ACCCTATAGAGGAGCATAGGAAG‐3′, and the reverse primer 5′‐GGCTCTTTGTTTGTCCAGTTTC‐3′.

### 2.8 Western blot

Total cell protein was exacted using RIPA lysis buffer, containing protease and phosphatase inhibitors. BCA protein assay was used to determine protein concentration (Beyotime Institute of Biotechnology). The protein samples (20μg) of each group were fractionated by SDS‐PAGE, and then transferred to PVDF membranes, which were blocked with 5% non‐fat milk in TBS‐T (Tris‐buffered saline with 0.1% Tween‐20) for 1‐2 hours at room temperature. The membranes were incubated with primary antibodies against YAP, Bax, Bcl‐2 and β‐actin overnight at 4°C, after which they were washed in TBS‐T and exposed to the corresponding secondary antibodies (1:10 000) at room temperature for 1‐2 hours. Fluorescent signal was detected with a Tanon 5100 imaging system, and quantified using Image J.

### 2.9 Immunofluorescence staining

Immunofluorescence staining for YAP in cells was performed using anti‐YAP antibody in a humidified box overnight at 4°C, followed by incubation with a fluorescein‐labelled secondary antibody for 1 hour at 37°C. Cell nuclei were stained with DAPI (5.0 μg/mL) for 5 minutes. The resulting immune‐stained samples were imaged by fluorescence microscopy (Olympus) at 200× magnification.

### 2.10 In vivo animal study

Male C57BL/6 mice, weighing 20‐25 g at 8 weeks of age, were obtained from the Experimental Animal Center at the Second Affiliated Hospital of Harbin Medical University (Harbin, China). All experimental procedures were performed in accordance with PR China Legislation Regarding the Use and Care of Laboratory Animals, and all experiments involving animals were approved by the Animal Care and Use Committee of the Second Affiliated Hospital of Harbin Medical University. The mice were kept in a pathogen‐free environment at 20 ± 5°C on a 12/12 hours dark/light cycle with free food and water access. The mice were randomly assigned to 4 groups (each group n = 10): (i) Control (Normal saline); (ii) Dox treatment (Dox); (iii) Mel treatment (Mel); (iv) Mel and Dox co‐treatment (Mel+Dox). Dox was intraperitoneally injected at a dose of 5 mg/kg per week for 5 weeks (for a 25 mg/kg cumulative dose). Mel (10 mg/kg) was administered intraperitoneally 24 hours before Dox injection.^22^, ^23^ All mice were anaesthetized with an intraperitoneal injection of 100 mg/kg sodium pentobarbital, followed by cervical dislocation 1 week after echocardiography examination.

### 2.11 Echocardiography

One week before initiation, as well as 7 days after the final injection of Dox, mice were anaesthetized with 1.5% isoflurane. Two‐dimensional and M‐mode echocardiographic measurements were carried out with a high‐resolution in vivo imaging system (VIVID E9, GE, USA). Left ventricular ejection fraction (LVEF), left ventricular fractional shortening (LVFS), along with left ventricular end diameter during diastole (LVEDd) and systole (LVESd), left ventricular posterior wall thickness during diastole (LVPWTd) and heart rate (HR) were measured. Short‐axis views were obtained from the parasternal approach. LV dimensions (LVEDd and LVESd) were measured in M‐mode. Ejection fraction was calculated as follows: (LVEDd^3^ − LVESd^3^)/LVEDd^3^ × 100, while fractional shortening was calculated as follows: (LVEDd − LVESd)/LVEDd × 100.

### 2.12 Haematoxylin‐Eosin staining (H&E staining)

Hearts were excised from anaesthetized mice, and the left ventricle was bisected along the long axis. One of the two parts was fixed overnight in 4% paraformaldehyde for paraffin embedding, while the other half was frozen in liquid nitrogen, followed by storage in −80°C. Formalin‐fixed, paraffin‐embedded myocardial tissue sections (5 μm thick) were deparaffinized, rehydrated and stained with haematoxylin and eosin (H&E). Morphological changes in myocardial tissues were observed under a light microscope.

### 2.13 Masson's trichrome staining

Masson's trichrome staining (Solarbio) was performed to measure cardiac collagen fraction. Briefly, paraffin‐embedded heart tissues were cut into 5 μm serial sections and placed on slides. Following deparaffinization and rehydration, sections were stained with standard Masson trichrome to analyse myocardial fibrosis and collagen fibre density. After staining, the normal myofibre was stained red, and the collagen was stained blue. The collagen fraction was defined as the blue‐stained area divided by the total field.

### 2.14 Immunohistochemical staining

Formalin‐fixed, paraffin‐embedded myocardial tissue sections (5 μm thick) were deparaffinized, rehydrated, washed in PBS and primary antibody applied overnight at 4°C. Thereafter, slides were incubated with peroxidase‐conjugated second antibody, diluted 1:100 in PBS, for 30 minutes. After washing in PBS, colouring reaction was carried out.

### 2.15 Statistical analyses

Data are presented as mean ± SD. SPSS 19.0 (SPSS Inc, Chicago, USA) software was used for data analysis. Student's *t*‐test was used for two‐group comparisons, while one‐way analysis of variance (ANOVA) was used for comparison among 3 or more groups, followed by Bonferroni post hoc tests. Differences were considered statistically significant at *P* < .05.

## 3 RESULTS

### 3.1 Mel treatment attenuated DOX‐induced mitochondrial oxidative stress and cytotoxicity

**Figure 1:**
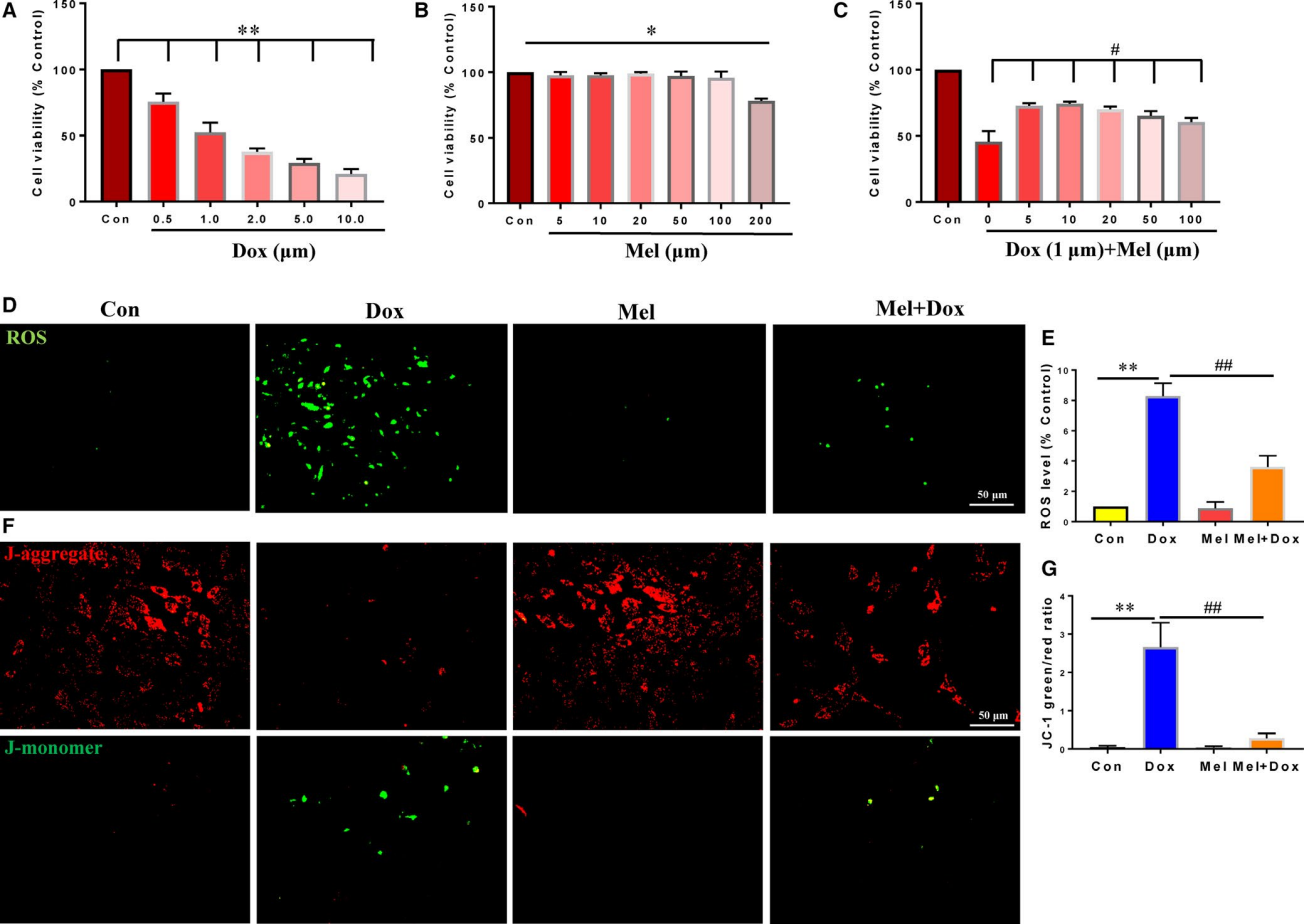
Mel treatment reversed the effects of Dox on H9c2 cells. Cell viability in control, as well as differing doses of doxorubicin (Dox, A), melatonin (Mel, B) and co‐treated Mel with Dox (C) in H9c2 cells. (D‐E): Representative fluorescent images of DCFH‐DA loading (D) and quantification of intracellular reactive oxidative species (ROS) levels in control, Dox, Mel and Mel+Dox treated H9c2 cells. Representative JC‐1 images (F) and quantification of JC‐1 green/red fluorescent ratio in the 4 groups of treated cells. n = 3 independent experiments/group. *P < .05 compared with the control group, **P < .01 compared with the control group, ^#^ *P* < .05 compared with the Dox‐treated group, ^##^ *P* < .05 compared with the Dox‐treated group

To examine the toxicity of Dox and Mel on H9c2 cells, CCK‐8 assay was employed to evaluate cell viability. The results demonstrated that Dox led to a dose‐dependent decrease in cell viability, where 1 μmol/L Dox treatment for 24 hours reduced H9c2 cell viability to 52% of its treatment value (Figure 1A). It has been suggested that 1 μmol/L concentration of Dox was comparative to the clinically relevant dose; thus, 1 μmol/L Dox was used in further experiments.^24^, ^25^ Mel concentrations between 5 and 100 μmol/L showed no cytotoxic effects, though a higher concentration of 200 μmol/L slightly reduced H9c2 cell viability (Figure 1B). To determine the appropriate concentration of Mel for treatment of Dox cardiotoxicity, different concentrations of Mel was used 24 hours prior to Dox treatment. Treatment with Mel significantly increased cell viability. In fact, subsequent experiments used 10 μmol/L Mel, as it demonstrated the highest viability (Figure 1C).

Reactive oxygen species is an indicator of mitochondrial oxidative stress and is responsible for cardiac dysfunction under various pathological conditions. Therefore, we used DCFH‐DA loading of H9c2 cells to detect mitochondrial ROS levels. Compared with either the control or Mel‐treated group, intracellular ROS levels were markedly increased under Dox. However, treatment with 10 μmol/L Mel decreased ROS level compared with the Dox‐only treated group (Figure 1D,E). Mitochondrial membrane potential (MMP) is an important factor for maintaining normal oxidative phosphorylation, whose decline indicates mitochondrial dysfunction and apoptosis. JC‐1 assay was used to measure MMP, where its staining manifests either as red fluorescence for normal, or green when MMP decreases. Dox‐treated H9c2 cells showed lessened red fluorescence and greater green fluorescence intensities, suggesting MMP decrease, whereas red fluorescence maintained the same intensity for Mel‐treated cells. The JC‐1 green/red fluorescent ratio was markedly decreased under treatment with Mel for 24 hours, indicating that it partially reversed oxidative stress level and relieved mitochondrial dysfunction of H9c2 cells caused by Dox (Figure 1F,G).

**Figure 2:**
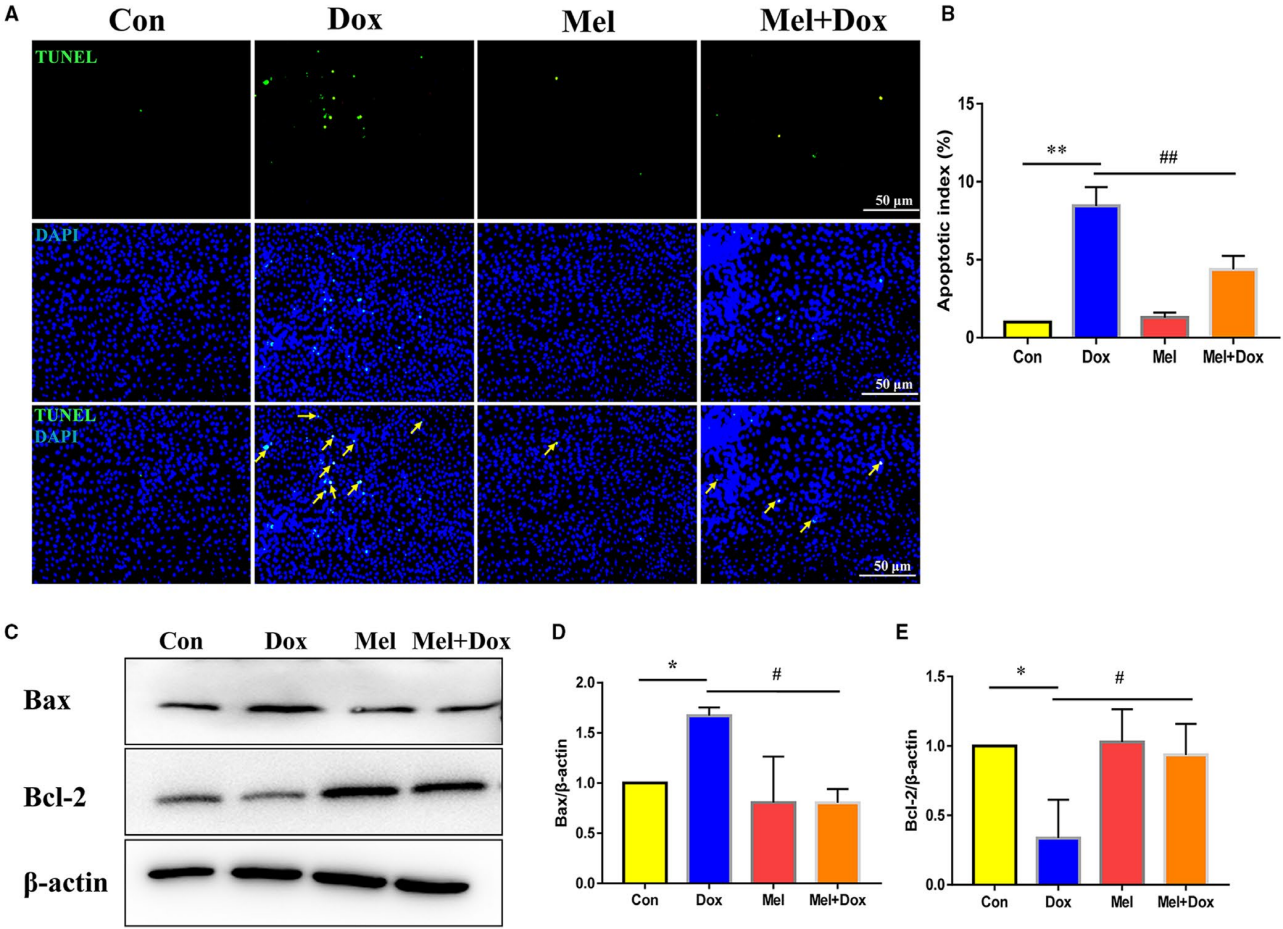
Mel alleviated Dox‐induced apoptosis in H9c2 cells. Representative images of TUNEL staining (A) and quantification of apoptosis (B) in control, doxorubicin (Dox), melatonin (Mel) and Mel with Dox co‐treated H9c2 cells. Representative Western blot images (C), quantification of Bax (D) and Bcl‐2 (E) in the 4 groups of treated cells. β‐actin was used as a house‐keeping protein. n = 3 independent experiments/group. *P < .05 compared with the control group, **P < .01 compared with the control group, ^#^ *P* < .05 compared with the Dox‐treated group, ^##^ *P* < .05 compared with the Dox‐treated group

Next, TUNEL staining was performed to determine cell apoptosis, where the results showed a significant increase in the number of apoptotic cells after Dox treatment. However, treatment with Mel for 24 hours largely decreased TUNEL‐positive cells in comparison with the Dox‐only treated group (Figure 2A,B). Western blot analysis of pro‐apoptotic Bax and anti‐apoptotic Bcl‐2 protein levels revealed that Dox significantly increased Bax and decreased Bcl‐2, reconfirming its causative role in cardiomyocyte apoptosis. By contrast, treatment with Mel for 24 hours significantly down‐regulated Bax and up‐regulated Bcl‐2 levels, compared to the Dox‐only group, showing that Mel protected cardiomyocytes from Dox‐induced apoptosis (Figure 2C‐E).

### 3.2 Mel treatment reversed the down‐regulation of YAP caused by Dox

**Figure 3:**
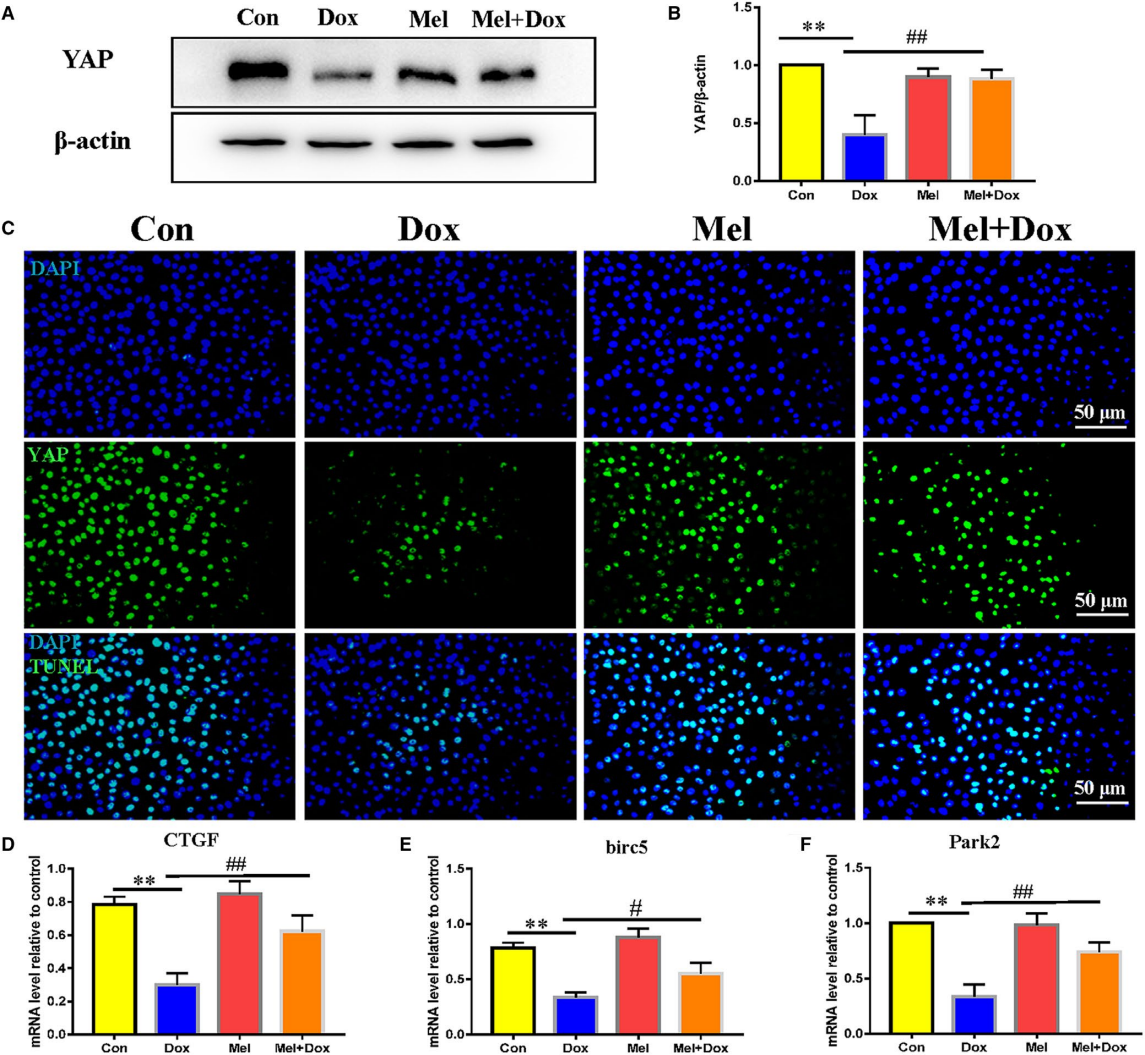
Effect of Mel on the expression of YAP in Dox‐treated H9c2 cells. Representative Western blot images (A) and quantification of Yes‐associated protein 1 (YAP) expression (B) in control, doxorubicin (Dox), melatonin (Mel) and Mel with Dox co‐treated H9c2 cells. (C) Representative fluorescent images showing YAP expression in the 4 groups of the H9c2 cells. Real‐time qPCR quantification of YAP target genes: Connective tissue growth factor (CTGF, D), baculoviral IAP repeat containing 5 (birc5, E) and parkin RBR E3 ubiquitin protein ligase (Park2, F) in the 4 groups of the cells. β‐actin was used as a house‐keeping protein. n = 3 independent experiments/group. *P < .05 compared with the control group, **P < .01 compared with the control group, ^#^ *P* < .05 compared with the Dox‐treated group, ^##^ *P* < .05 compared with the Dox‐treated group

To explore the possible mediators responsible for Dox‐induced cytotoxicity, we investigated YAP expression in treated H9c2 cells. Western blot analysis showed that YAP expression levels were significantly decreased after Dox treatment, which was restored back to the control level with treatment of Mel (Figure 3A,B). Immunofluorescence staining showed that YAP was mostly localized in the nuclei of H9c2 cells in both control and Mel treatment groups. Dox significantly decreased nuclear YAP expression, whereas Mel treatment preserved that expression there (Figure 3C).

CTGF, Park2 and Birc5 are target genes of YAP. RT‐qPCR was used to quantify the mRNA levels of these genes, where they were all found to be significantly reduced in Dox‐treated H9c2 cells. Mel treatment partially recovered CTGF, Park2 and Birc5 expression (Figure 3D‐F).

All the above evidence suggested that Dox treatment caused down‐regulation of YAP and its target genes, which Mel could partially rescue.

### 3.3 Mel protected H9c2 cells from Dox‐induced cytotoxicity through restoration of YAP expression

**Figure 4:**
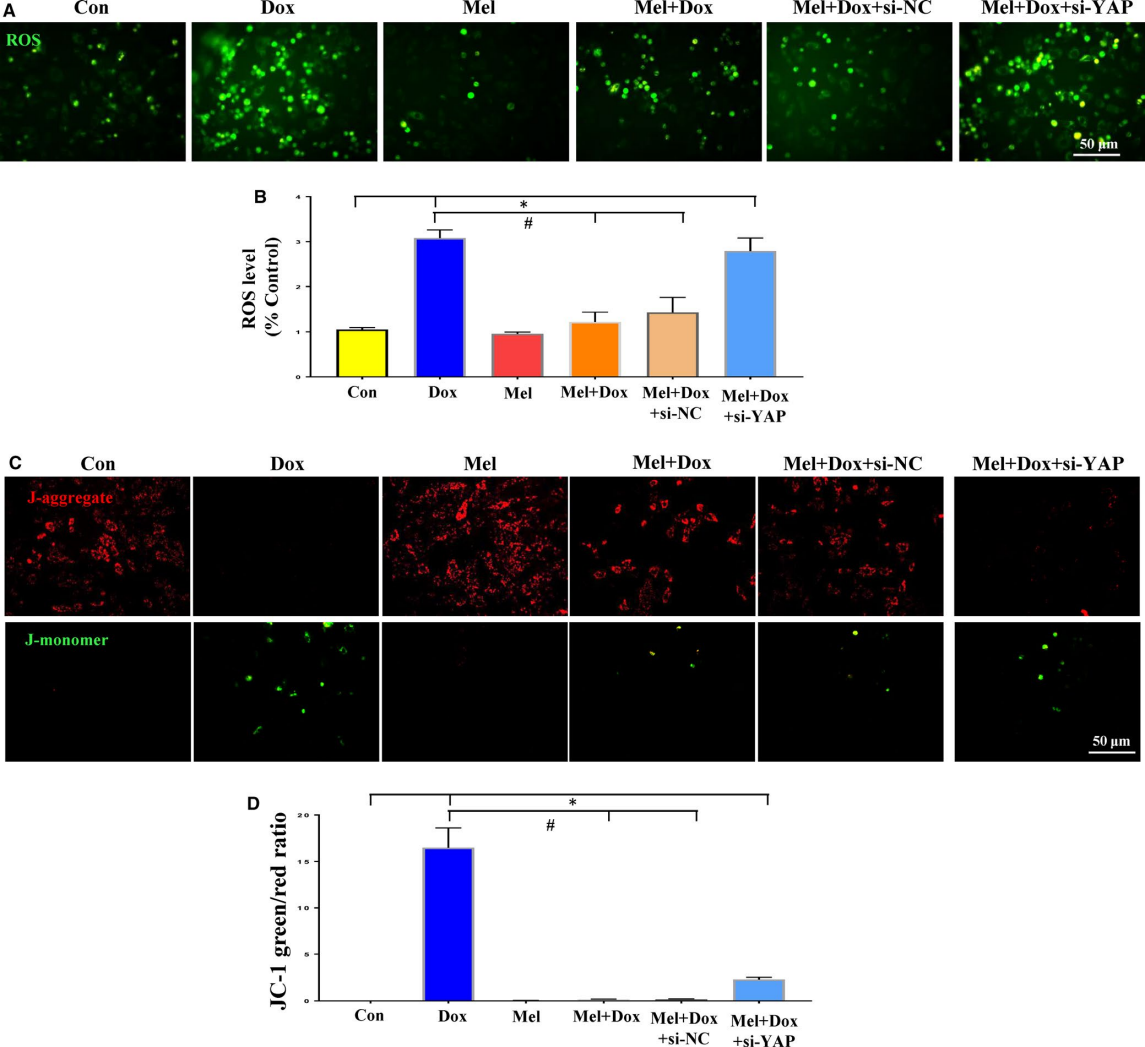
Knockdown of YAP abolished the protective effects of Mel on Dox‐induced cytotoxicity. DCFH‐DA loading (A) and quantification of intracellular reactive oxidative species (ROS, B) levels in control, doxorubicin (Dox), melatonin (Mel), Mel+Dox, Mel+Dox+siNC (control siRNA) and Mel+Dox+siYAP treated H9c2 cells. Representative JC‐1 images (C) and quantification of JC‐1 green/red fluorescent ratio (D) in the 6 groups of treated cells. β‐actin was used as a house‐keeping protein. n = 3 independent experiments/group, *P < .05 compared with the control group, **P < .01 compared with the control group, ^#^ *P* < .05 compared with the Dox‐treated group, ^##^ *P* < .05 compared with the Dox‐treated group

**Figure 5:**
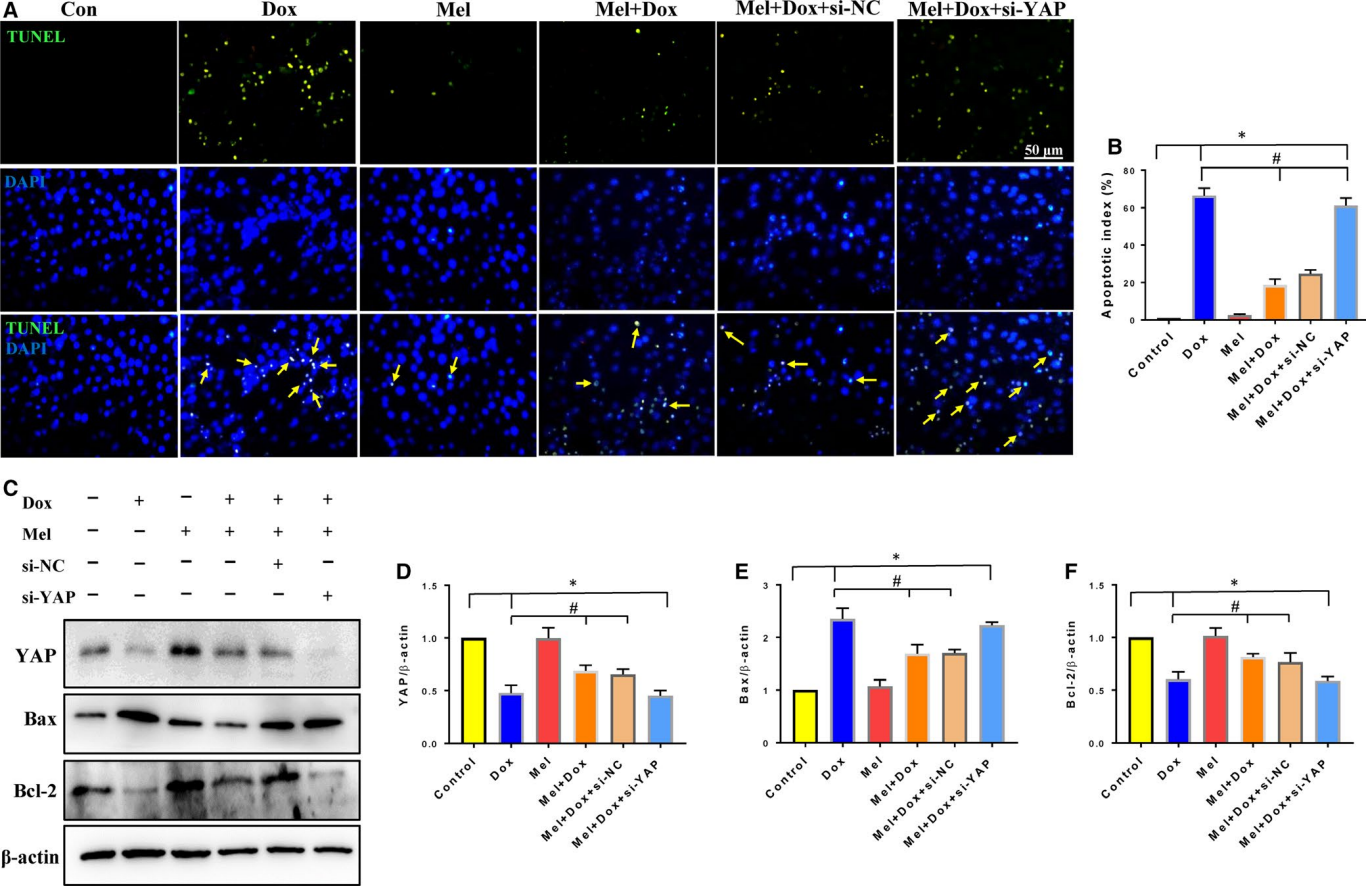
Knockdown of YAP reversed the protective effect of Mel on Dox‐ induced apoptosis. Representative images of TUNEL staining (A) and quantification of apoptosis (B) in the control, doxorubicin (Dox), melatonin (Mel), Mel+Dox, Mel+Dox+siNC (control siRNA) and Mel+Dox+siYAP treated H9c2 cells. Representative Western blot images (C) and quantification of Yes‐associated protein 1 (YAP, D), Bax (E) and Bcl‐2 (F) are shown. β‐actin was used as a house‐keeping protein. n = 3 independent experiments/group, *P < .05 compared with the control group, **P < .01 compared with the control group, ^#^ *P* < .05 compared with the Dox‐treated group, ^##^ *P* < .05 compared with the Dox‐treated group

To confirm the causal relationship between YAP and Mel in treating Dox‐induced cardiotoxicity, we down‐regulated YAP level with siRNA. Western blot analysis showed that YAP expression in H9c2 cells was efficiently knocked down by YAP‐targeted siRNA (Figure S1A,B). Although Mel treatment significantly reduced the elevated ROS level caused by Dox, it was reversed when Mel treatment was combined with YAP knockdown (Figure 4A,B). Furthermore, this combination of Mel treatment and YAP knockdown markedly decreased MMP, as indicted by increased JC‐1 green/red fluorescent ratio (Figure 4C,D). Accordingly, YAP knockdown reversed the decreased number of TUNEL‐positive cells under Mel treatment (Figure 5A,B). Western blot results were also consistent with the above data from the other assays, showing that knockdown of YAP impaired the anti‐apoptotic effect of Mel, by increasing Bax and decreasing Bcl‐2 expression (Figure 5C‐F). Our results proved that Mel exerted its protective role on Dox‐induced cardiotoxicity through restoring YAP expression.

### 3.4 Mel alleviated Dox‐induced myocardial injury by restoring YAP expression

**Figure 6:**
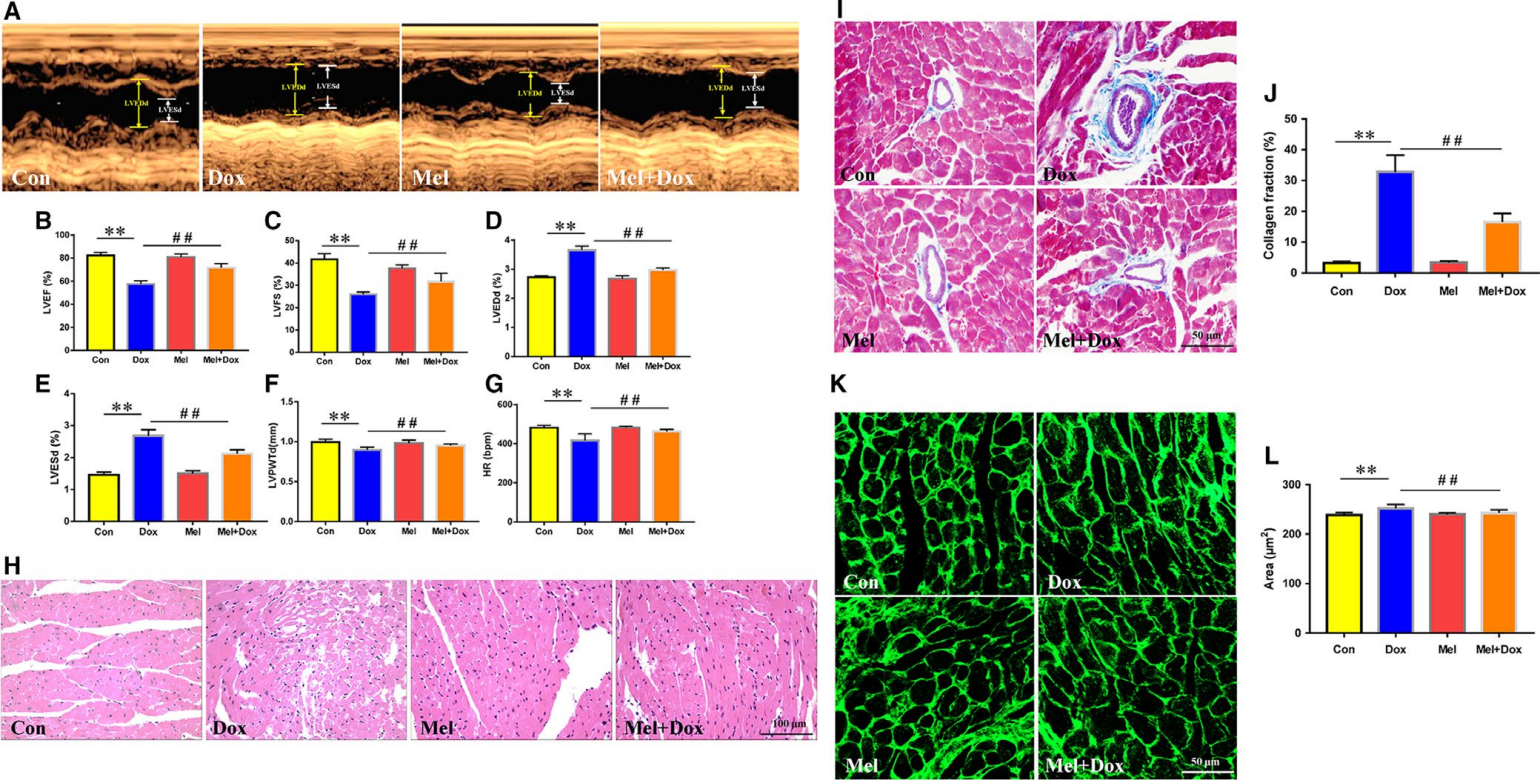
Effect of Mel on Dox‐induced cardiac toxicity and dysfunction in mouse hearts. Representative M‐mode echocardiographic images (A) of control, doxorubicin (Dox), melatonin (Mel) and Mel+Dox treated mouse hearts. Quantitative analyses of left ventricular ejection fraction (LVEF, B), left ventricular fractional shortening (LVFS, C), left ventricular end diameter during diastole (LVEDd, D) and systole (LVESd, E), left ventricular posterior wall thickness during diastole(LVPWTd, F) and heart rate(HR, G) are, respectively, presented. (H) Representative H&E staining of the sectioned left ventricle from the 4 groups of animals. (I, J) The myocardial fibrosis was determined by Masson's Trichrome staining and the collagen fraction was calculated. (K, L) Wheat germ agglutinin (WGA) staining was used to evaluated changes in cardiomyocyte size. n = 6/group, *P < .05 compared with the control group, **P < .01 compared with the control group, ^#^ *P* < .05 compared with the Dox‐treated group, ^##^ *P* < .05 compared with the Dox‐treated group

To investigate whether the in vitro finding also holds true in the in vivo cardiac injury model, we treated C57BL/6 mice with Dox and/or Mel for 5 weeks and performed echocardiographs afterwards to evaluate cardiac function (Figure 6A). LVEF (Figure 6B), LVFS (Figure 6C), LVPWTd (Figure 6F) and HR (Figure 6G)were found to be significantly decreased in Dox‐treated mice compared to controls. In addition, left ventricular remodelling was noted in the Dox group, as evidenced by dilated LVEDd (Figure 6D) and LVESd (Figure 6E). However, the Mel and Dox co‐treated group yielded increased LVEF, LVFS, LVPWTd and HR, and significantly decreased LVEDd and LVESd, compared to Dox‐only mice (Figure 6A‐G).

**Figure 7:**
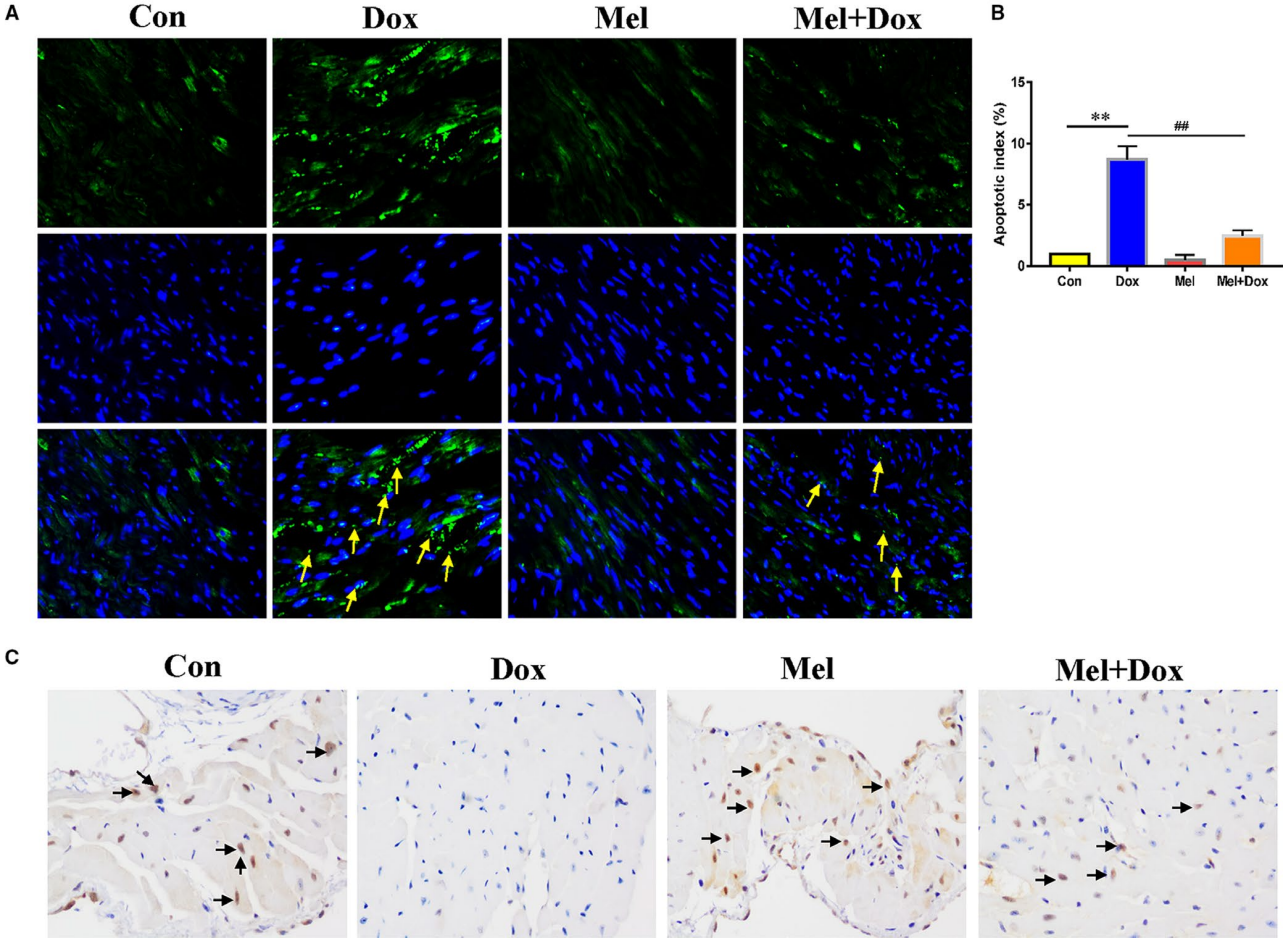
Mel co‐treatment with Dox decreased apoptosis and preserved YAP level in mouse hearts. Representative images of TUNEL staining (A) were obtained, and apoptosis index (B) was calculated from control, doxorubicin (Dox), melatonin (Mel) and Mel+Dox treated mouse hearts. (C) Representative immunohistochemical staining of Yes‐associated protein 1 (YAP) in the sectioned left ventricle from the 4 groups of animals. n = 6/group, *P < .05 compared with the control group, **P < .01 compared with the control group, ^#^ *P* < .05 compared with the Dox‐treated group, ^##^ *P* < .05 compared with the Dox‐treated group

Histological examination revealed visible myocardial damage in Dox‐treated animals. H&E staining showed that the Dox‐only mice had cardiomyocyte dysplasia, vacuolar degeneration and interstitial oedema (Figure 6H). All those conditions were found to have lower occurrences in the Mel and Dox co‐treatment group (Figure 6H). Masson's Trichrome staining revealed that the Dox significantly increased cardiac collagen fraction, which was decreased by Mel treatment (Figure 6I,J). Wheat germ agglutinin (WGA) staining was used to visualize the myocyte membranes and measure the cardiomyocyte area. The results showed that the cardiomyocyte size was increased in the Dox‐treated group and dropped to nearly normal level with Mel co‐treatment (Figure 6K,L). TUNEL staining showed that Dox‐treated mice had significantly higher number of apoptotic cells, compared to the control group (Figure 7A,B). However, Mel and Dox co‐treatment significantly decreased TUNEL‐positive cells in mouse heart tissue in comparison with the Dox‐only group (Figure 7A,B). Immunohistochemistry staining of YAP revealed that five weeks of Dox treatment notably decreased YAP‐positive areas when compared to the control group, while the combinational treatment of Mel and Dox preserved the expression of YAP, compared to the Dox‐only group (Figure 7C).

All in vivo evidence was in agreement with our in vitro findings, suggesting that Mel exerted myocardial protection against DOX‐induced cytotoxicity via the preservation of the Hippo/YAP signalling pathway.

## 4 DISCUSSION

Dox is an effective anticancer drug, which has been widely used for the treatment of solid and haematologic malignancies over the past decades. On the other hand, it can cause dose‐dependent cardiac injury, which eventually leads to cardiomyopathy and heart failure. In this study, we confirmed in vitro and in vivo observation of cardiotoxicity caused by Dox. In vitro, cell viability of H9c2 cells was decreased by Dox treatment, which was accompanied by aggravated ROS production and cardiomyocyte apoptosis. However, treatment with Mel reversed the above damage. In vivo, a cumulative dose of 25 mg/kg of Dox caused obvious cardiac tissue injury, as demonstrated by echocardiography, H&E staining, Masson's Trichrome staining and WGA staining, yielding decreased cardiac function, pathological and myocytes changes, as well as collagen increase. Co‐treatment of Mel with Dox partially preserved cardiac function, tissue morphology and collagen fraction. YAP is an important transcriptional co‐activator, as well as the effector of the Hippo pathway, where it participates in diverse cardiac physiological and pathological processes, including development, apoptosis, hypertrophy, autophagy, angiogenesis and cardiomyocyte regeneration.^17^, ^26^, ^27^, ^28^ The results of this research demonstrated that Dox significantly decreased the expression of YAP, while Mel co‐treatment partially restored the expression of YAP. Additionally, down‐regulating the expression of YAP by siRNA abolished the protective effect of Mel on Dox‐induced pathological changes. Taken together, our results suggested that Mel protects cardiomyocytes from Dox‐induced injury by maintaining YAP levels.

The mechanisms underlying anthracycline‐induced cardiotoxicity have been investigated for decades. Among the many mechanisms proposed, excess free radical production, with membrane lipid peroxidation and mitochondrial dysfunction, is most widely accepted.^1^ Mitochondria account for 45% of the myocardial volume and play a pivotal role in energy production, maintaining homoeostasis, metabolism regulation, mitophagy and apoptosis.^4^ Therefore, cardiac tissue with enriched mitochondrial content is more susceptible to anthracyclines, such as Dox, inducing cytotoxicity. It has been reported that Dox treatment causes increased ROS and lower MMP levels.^2^, ^29^ The results of this experiment displayed a significant up‐regulation of ROS levels in Dox‐treated H9c2 cells and mouse hearts, along with MMP decreases. Our results were consistent with previous findings showing Dox targeting mitochondria and causing oxidative stress damage.

Recent research suggests that Mel plays an important role in various cardiovascular diseases, including ischaemia‐reperfusion injury, atherosclerosis, hypertension, heart failure and drug‐induced myocardial injury.^11^, ^30^, ^31^ Mel protects mitochondria from ischaemia‐reperfusion injury by preserving mitochondrial structure integrity and promoting ATP synthesis.^32^ In addition, Zhu et al^33^ showed that Mel improved the survival and function of implanted adipose‐derived mesenchymal stem cells from MI, via increasing the expression of Cu/Zn superoxide dismutase and inhibiting the activation of the caspase cascade. Mel has also been demonstrated to reduce the expression of apoptotic proteins and increase the anti‐apoptotic protein production, to relieve hypoxia/serum deprivation‐induced injury.^34^ In agreement with previous reports, the present study found that ROS level was significantly reduced in Mel treatment compared with cardiomyocytes only treated with Dox. Mel efficiently re‐established MMP, as manifested by higher red in comparison with green fluorescence. As a result, Mel protected cardiomyocytes from Dox‐induced apoptosis through decreasing apoptosis‐promoting protein Bax and increasing apoptosis‐suppressing protein Bcl‐2 expression. The aforementioned results indicated that Mel exerted cardioprotective effects against Dox via inhibiting oxidative stress injury and apoptosis.

The Hippo signalling pathway, first discovered in *Drosophila melanogaster*, plays a pivotal role in cardiovascular development, growth, homoeostasis, disease and regeneration. With respect to its development, YAP‐conditioned knockout embryos had an abnormally thin myocardium 35 and lethal cardiac hypoplasia with low proliferative ability.^36^ Singh et al^37^, employing both genetic and pharmacological approaches, were able to demonstrate that inhibition of Hippo signalling mediators YAP and TAZ leads to impaired epicardial epithelial‐to‐mesenchymal transition and reduced epicardial cell proliferation and differentiation into coronary endothelial cells. Besides cardiovascular development, Hippo/YAP pathway has been investigated for its involvement in the pathogenesis of hypertrophic cardiomyopathy (HCM). It is demonstrated that up‐regulation of YAP occurs in both HCM patient samples and transverse aortic constriction murine models, as well as decreased phosphorylation of YAP at serine 127, accompanied by increased transcription of YAP mediated genes.^38^ Chen et al^39^ delineated that the Hippo pathway is activated and is behind the occurrence of adipogenesis in arrhythmogenic cardiomyopathy. Hippo signalling pathway also plays a critical role in cardiac ischaemic injury and MI, where YAP exerts pro‐survival effect on cardiac tissue through interaction with FoxOs, miR‐206 and Akt.^40^ Cardiac‐specific YAP heterozygous knockout mice show significant increases in apoptosis after MI.^18^ Inhibition of phosphorylated YAP expression by deleting SAV1, MST1/2 or LATS2 results in obvious cardiomegaly.^27^ Cardiac‐specific overexpression of YAP, or inhibition of phosphorylated YAP, exerts beneficial effect on cardiac repair after MI and ischaemia‐reperfusion injury.^18^, ^20^, ^35^, ^41^ Luo et al^42^ indicated that YAP level is reduced in a concentration‐dependent manner after treatment with Dox, and overexpression of YAP significantly reduces c‐PARP and c‐Caspase‐3 expression levels, which are positively correlated with apoptosis. The current study also showed that YAP expression decreased significantly after Dox treatment in H9c2 cells. Immunohistochemical experiments revealed similar results after Dox treatment in mouse hearts. However, Mel treatment in cells, and co‐treatment with Dox in mice, restored YAP expression close to the control level, along with reduced Bax and increased Bcl‐2 expression. By contrast, knockdown of YAP by siRNA abolished the above‐mentioned protective effects of Mel, via aggravating oxidative stress and apoptosis in Dox‐treated H9c2 cells. All these findings confirmed the role of YAP as a key factor responsible for Med‐mediated protection against Dox‐induced cardiac toxicity.

The significance of the outcome of Mel treatment in cancer patients with Dox treatment should be considered. This is due to Dox acting in the context of ROS production, where Mel treatment serves as a free radical scavenger meaning that Mel could contra‐indicate the use of Dox in such patients. However, past evidence indicated that despite having potent antioxidant and anti‐apoptotic actions in normal cells, Mel in many cancer types, including breast cancers, have pro‐oxidant, anti‐proliferative, anti‐angiogenic and immunomodulatory properties. Indeed, research has shown when Mel was used as an adjuvant for arsenic trioxide treatment, it inhibits the growth of breast cancer cells via inducing p21 expression, leading to G1 cell cycle arrest and suppression of human telomerase reverse transcriptase and proto‐oncogene Myc protein.^43^ Additionally, a new study has shown that Mel treatment effectively down‐regulated miR‐24, an important oncogenic miRNA which reduces the activity of the p38‐p53 axis components. Those components, in turn, are involved in DNA repair and inhibition of cell proliferation in breast cancers.^44^ It is plausible to reason that apart from its cardioprotective effect on Dox, Mel may act in synergy with Dox to counteract tumour growth, making it an ideal adjuvant for antitumour medication.

To conclude, our results revealed that Mel protected cardiomyocyte viability in vitro, and cardiac tissue against Dox‐induced cardiotoxicity in vivo, through reduced oxidative stress injury and apoptosis. YAP is a key signalling molecule involved in this process, whose preservation by Mel treatment reduced oxidative stress injury and maintained cardiac tissue morphology. To our knowledge, this is the first study to report the relationship between Mel treatment and Hippo/YAP pathway in Dox‐induced cardiotoxicity. These findings will provide valuable information for mechanism behind, as well as the prevention of, Dox‐induced cardiotoxicity.

## CONFLICT OF INTEREST

The authors declare that they have no conflict of interest.

## AUTHOR CONTRIBUTIONS

Hai‐ru Li performed the cytological experiments, analysed the data and wrote the paper. Chao Wang was responsible for doxorubicin and melatonin administration and echocardiography examination. Chao Wang, Ping Sun and Dan‐dan Liu performed the molecular biology experiments. Experiment design, calibration and supervision, as well as final approval, were done by Jia‐wei Tian and Guo‐qing Du.

## Data Availability

The datasets analysed during the current study are available from the corresponding author upon reasonable request.
