# Corrigendum

**DOI:** 10.1111/jcmm.16056

**Published:** 2021-01-15

In Li et al,^1^ the published article contains errors in Figures 3 and 6. The correct figures are shown below. The authors confirm all results and conclusions of this article remain unchanged.

**Figure 3:**
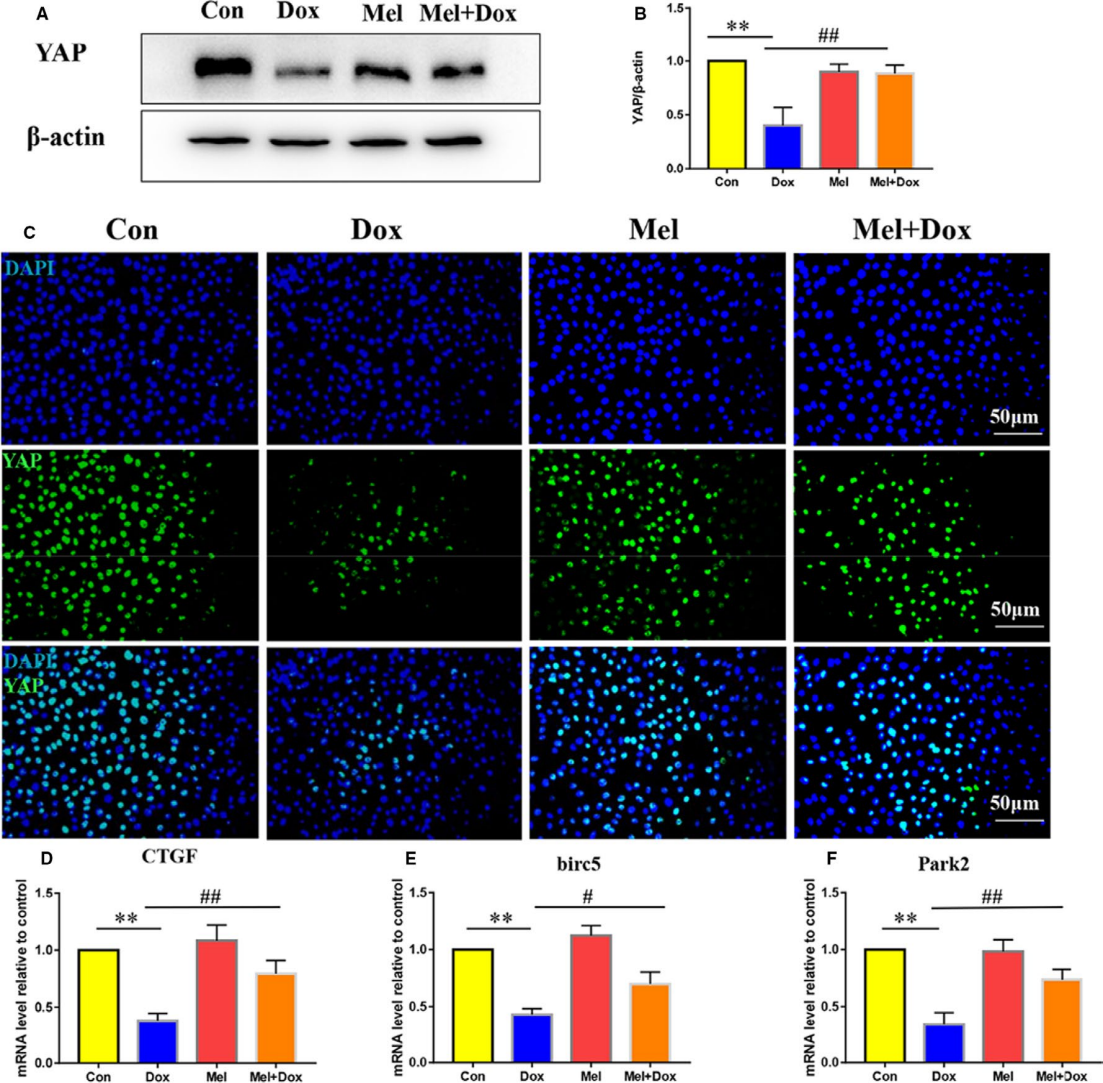
Effect of Mel on the expression of YAP in Dox‐treated H9c2 cells. Representative Western blot images (A) and quantification of Yes‐associated protein 1 (YAP) expression (B) in control, doxorubicin (Dox), melatonin (Mel) and Mel with Dox co‐treated H9c2 cells. (C) Representative fluorescent images showing YAP expression in the 4 groups of the H9c2 cells. Real‐time qPCR quantification of YAP target genes: Connective tissue growth factor (CTGF, D), baculoviral IAP repeat containing 5 (birc5, E) and parkin RBR E3 ubiquitin protein ligase (Park2, F) in the 4 groups of the cells. β‐actin was used as a house‐keeping protein. n = 3 independent experiments/group. *P < .05 compared with the control group, **P < .01 compared with the control group, #*P* < .05 compared with the Dox‐treated group, ##*P* < .05 compared with the Dox‐treated group

**Figure 6:**
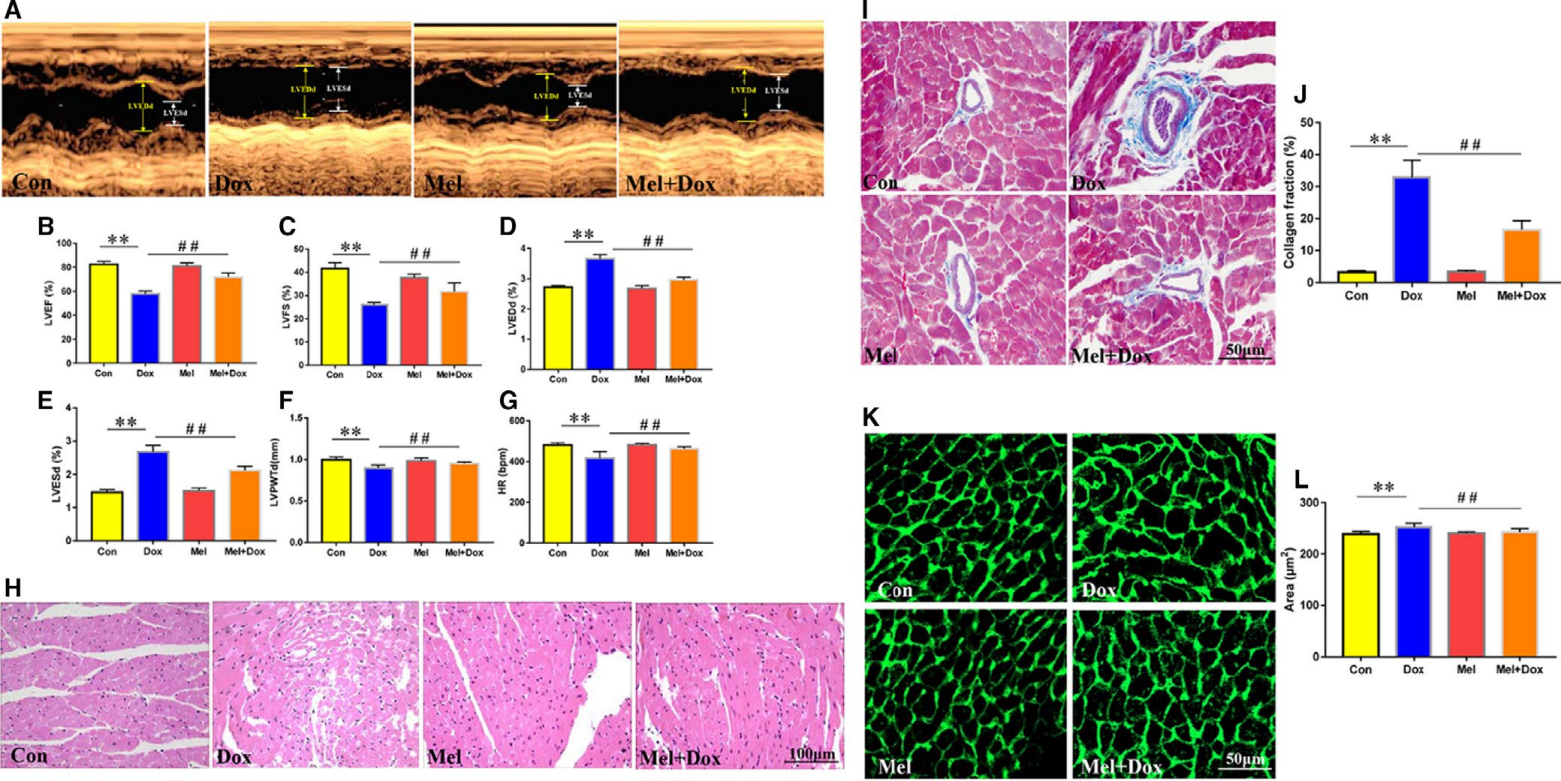
Effect of Mel on Dox‐induced cardiac toxicity and dysfunction in mouse hearts. Representative M‐mode echocardiographic images (A) of control, doxorubicin (Dox), melatonin (Mel) and Mel+Dox treated mouse hearts. Quantitative analyses of left ventricular ejection fraction (LVEF, B), left ventricular fractional shortening (LVFS, C), left ventricular end diameter during diastole (LVEDd, D) and systole (LVESd, E), left ventricular posterior wall thickness during diastole (LVPWTd, F) and heart rate (HR, G) are, respectively, presented. (H) Representative H&E staining of the sectioned left ventricle from the 4 groups of animals. (I, J) The myocardial fibrosis was determined by Masson's Trichrome staining, and the collagen fraction was calculated. (K, L) Wheat germ agglutinin (WGA) staining was used to evaluated changes in cardiomyocyte size. n = 6/group, *P < .05 compared with the control group, **P < .01 compared with the control group, #*P* < .05 compared with the Dox‐treated group, ##*P* < .05 compared with the Dox‐treated group
