# Supplementary figures and images for: Melatonin attenuates doxorubicin‐induced cardiotoxicity through preservation of YAP expression
Source: J Cell Mol Med. 2020 Feb 18;24(6):3634–46. doi: 10.1111/jcmm.15057 (PMC7131936; doi:10.1111/jcmm.15057)

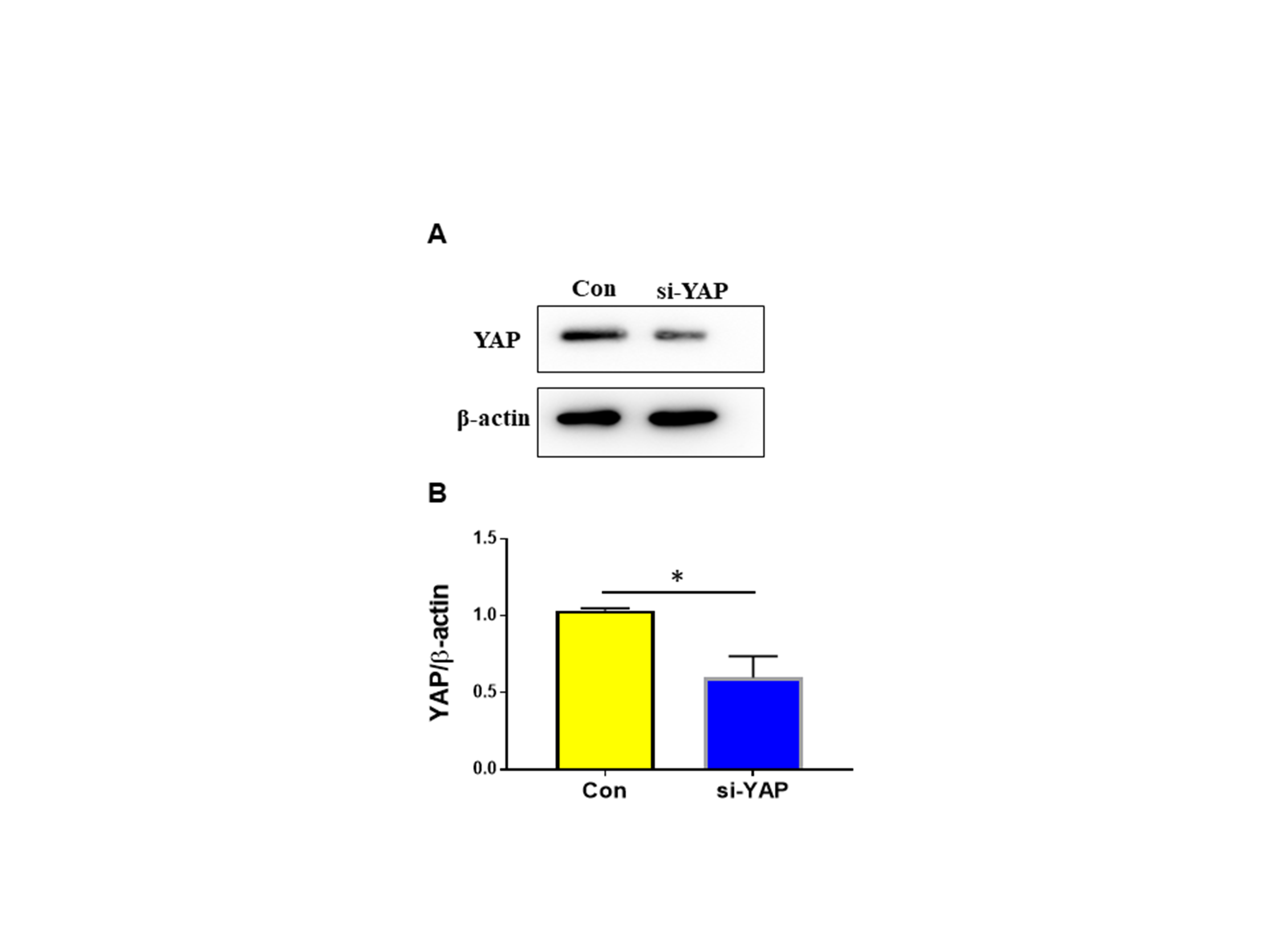

Supplement: Supplementary file 1 [file JCMM-24-3634-s001.tif]
